# Comparative Genomics and Characterisation of the Role of *Saccharomyces cerevisiae* Respiration in the Fermentation of Chinese Steamed Bread

**DOI:** 10.3390/jof11020114

**Published:** 2025-02-03

**Authors:** Yawen Gao, Yufeng Guo, Jianing Pang, Mingkai Liu, Tengdan Yuan, Qinhong Wang, Jingsheng Liu

**Affiliations:** 1 College of Food Science and Engineering, Jilin Agricultural University, No. 2888 Xincheng Street, Jingyue District, Changchun 130118, China; gaoyawen@jlau.edu.cn (Y.G.);; 2 Key Laboratory of Engineering Biology for Low-Carbon Biosynthesis, Tianjin Institutes of Industrial Biotechnology, Chinese Academy of Sciences, 32 West 7th Avenue, Tianjin Airport Economic Area, Tianjin 300308, China; 3 National Center of Technology Innovation for Synthetic Biology, Tianjin 300308, China; 4 College of Food Science and Engineering, Qilu University of Technology (Shandong Academy of Science), Jinan 250353, China

**Keywords:** Chinese steamed bread, *Saccharomyces cerevisiae*, genomic analysis, phenotype, flavour substance

## Abstract

The genetic composition of *Saccharomyces cerevisiae* and its various phenotypes during fermentation significantly correlate to the quality of Chinese steamed bread (CSB). However, the systematic correlation between different *S. cerevisiae* and CSB has not been fully elucidated. Herein, we characterised CSBs prepared with 36 isolates of *S. cerevisiae* (designated S1–S36) to comparatively evaluate their correlations. CSBs 1, 2, 13, 21, 25 and 33 exhibited suitable total titratable acidity (TTA) values, pH values and large specific volumes. Texture analysis showed that CSBs 1, 25 and 33 exhibited higher springiness and cohesiveness values. CSBs 8, 25 and 33 exhibited low hardness, gumminess and chewiness values. At the micro level, CSBs 1, 25 and 33 showed a loose reticular structure with large holes and in which starch particles wrapped into gluten protein. Fifty-nine volatile flavour compounds belonging to six categories were determined in 10 selected CSBs, and CSBs 1, 25 and 33 contained more flavour and balanced substance categories. In addition, comparative genomic analysis revealed 33 non-synonymous mutations in the three strains with strong fermentation ability (S1, S25 and S33) and the three strains with weak fermentation ability (S18, S20 and S35) involving 19 genes, including: the respiration-related genes *COS5*, *COS8* and *COX10*; the starch metabolism transcription factor *MSS11*; the general transcription factor *SPT8*; the cell aggregation-related gene *FLO1* and the transporter gene *SEO1*. Other genes with different genotypes were also enriched in respiration-related gene ontology terms. These data offer preliminary experimental evidence regarding the application of *S. cerevisiae* S1, S25 and S33 in fermented foods derived from grains.

## 1. Introduction

Chinese steamed bread (CSB), which is made with wheat flour, water and yeast by fermentation, is a traditional staple food in China with a long history of consumption. Consumers primarily choose CSB based on two important factors: mouthfeel and flavour. However, the main factor affecting the mouthfeel and flavour of CSB is the starter [1,2]. Currently, *Saccharomyces cerevisiae* is the main starter used in CSB production. During the *S. cerevisiae* fermentation process, in addition to CO_2_ and ethanol, some by-products, such as low molecular weight organic acids, alcohols and esters are generated, and they have a positive effect on the volume, texture and flavour of CSB [3,4]. CO_2_ produced during yeast fermentation can increase the expansion of gluten, strengthening the gas-holding capacity of dough, thereby improving the volume and mouthfeel of CSB [5]. The types and quantities of aromatic substances produced by yeast cells during fermentation differ, and different yeasts also secrete different flavour substances, which would contribute to the flavour formation of CSB [6].

The effects of yeast on CSB quality are attributed to the genetic and phenotypic characteristics of the yeast involved in CSB fermentation. The genome of *S. cerevisiae* has been extensively studied due to its pivotal role in multiple fermentation industries, including beer, wine and dairy production. As a model organism and industrial microorganism, its genetic framework has been leveraged to enhance fermentation efficiency, flavour compound production and overall process optimization. *FLO1* and *FLO11* encode cell wall proteins responsible for flocculation, impacting yeast sedimentation and beer clarity during and after fermentation [7,8]. MAL genes (e.g., *MAL11*, *MAL31*) regulate maltose uptake and metabolism, critical for efficient sugar utilization in wort fermentation [9]. The HXT family genes encode hexose transporters, ensuring efficient glucose and fructose uptake under high sugar conditions in grape must [10]. ADH1 and ADH2 encode alcohol dehydrogenases essential for ethanol production [11,12].

In previous studies, researchers have predominantly concentrated on several pivotal issues, including the composition of yeast communities during dough fermentation and the enhancement of bread quality [13,14]. Viola et al. [15] explored effects of the interaction of *Saccharomyces cerevisiae*, *Leuconostoc mesenteroides* and *Papiliotrema terrestris* during dough fermentation on bread quality. Chiva et al. [16] characterized the phenotypic traits of selected yeast strains from mother dough, bakery dough, and a variety of raw cereal matrices from Spain, and analyzed the fungal microbiota in some mother dough. However, the correlation between the genetic and phenotypic characteristics of yeast *S. cerevisiae* and the quality of CSB that can be made via comparative genomics is rarely elucidated.

Therefore, this study aimed to determine the behaviour and contribution of 36 isolates of *S. cerevisiae* to CSB production. The total titratable acidity (TTA), specific volume, texture, microstructure and volatile compounds of CSB were investigated to evaluate the effects of different *S. cerevisiae* on the quality and flavour of CSB. In addition, comparative genomic analysis was performed on *S. cerevisiae* to determine the relationship between *S. cerevisiae* genotype and CSB quality. The findings of this study will serve as a preliminary data for the cultivation of *S. cerevisiae*, aimed at enhancing the quality of grain-based fermented foods.

## 2. Materials and Methods

### 2.1. Materials

Thirty-six isolates of *S. cerevisiae* (designated S1–S36) were kindly provided by the Tianjin Institute of Industrial Biotechnology, Chinese Academy of Sciences (Tianjin, China). Wheat flour was obtained from the COFCO Flour Industry (Bayannaoer) Co., Ltd. (Bayannaoer, China). The moisture, protein, lipid and carbohydrate contents of the flour were 13.8%, 10%, 1.5% and 74%, respectively, according to the manufacturer’s description.

### 2.2. Culture of Strains

The *S. cerevisiae* were cultured according to the method described by Shui et al. [17] with some modifications. A single colony was inoculated into 5 mL of yeast peptone dextrose (YPD) medium containing 20 g/L glucose and incubated at 30 °C and 200 rpm for 18 h. Next, the seed culture was inoculated into a 250-mL shaking flask containing 50 mL of YPD medium containing 100 g/L of glucose and incubated at 30 °C and 200 rpm for 48 h. After incubation, the yeast cells were harvested via centrifugation at 4 °C and 8000× *g* for 5 min. The precipitate was washed three times before diluting to a 100-fold dilution with distilled water. The cell density (OD_600_) was measured at a wavelength of 600 nm using a spectrophotometer (Unico (Shanghai) Instrument Co., Ltd., Shanghai, China).

### 2.3. Preparation of CSBs

The CSBs were prepared as described in a previous study with some modifications [18]. The formula for each CSB sample is 100 g of wheat flour, 50% water (flour weight basis) and 900 OD_600_ *S. cerevisiae* (S1–S36, respectively). The CSB samples corresponding to the utilized *S. cerevisiae* serial numbers (S1–S36) were designated as CSB1-CSB36, respectively, while the CSB samples devoid of added *S. cerevisiae* served as the control group. First, according to the formula, the ingredients were mixed by hand for 15 min to form a smooth dough. Second, the dough was fermented in an incubator (Model SHP-250, Shanghai Jing Hong Laboratory Instrument Co., Ltd., Shanghai, China) at 30 °C for 1 h with 85% relative humidity. Third, the dough was pressed 10 times using a noodle maker (Model DZM160, Zaoyang Hongchao Food Machinery Co., Ltd., Zaoyang, China) and then formed into a round shape by hand. Subsequently, the raw CSB billet was fermented in an incubator at 37 °C for 40 min at 80% relative humidity. Finally, the raw CSB billet was steamed for 20 min in a steamer and cooled at room temperature (25 ± 2 °C). For each strain, three CSBs were prepared for subsequent experiments.

### 2.4. Determination of pH and TTA

The pH values were measured using a pH-meter (FE20, Mettler Toledo Instruments (Shanghai) Co., Ltd., Shanghai, China). The TTA value was examined as described in a previous study with some modifications [19]. Briefly, 10 g of CSB was mixed with 50 mL of distilled water and titrated with 0.1-N NaOH to reach pH 7, and the consumed volume of NaOH (mL) was expressed. The analysis was conducted three times for each sample.

### 2.5. Specific Volume Evaluation

The volume of CSB was measured using the millet replacement method, and the specific volume was expressed as the ratio of the volume (mL) to the weight (g) [20]. The analysis was conducted three times for each sample.

### 2.6. Texture Profile Analysis

The texture profile of CSB was analysed as described in a previous study with some modifications [13]. A core of each CSB sample was excised and segmented into 2 cm × 2 cm × 1 cm cubes. The CSB sample was measured using a texture analyser (TA) (XT plus Texture Analyser, Stable Micro Systems Ltd., Godalming, UK), equipped with a 10 mm (3/8″) probe. The test conditions are described as follows: induction force = 1000 N, residence time = 5 s, probe back to the height of the sample surface 30 mm, test speed = 1.0 mm/s, strain deformation = 30% and minimum trigger force = 0.8 N. The hardness, springiness, gumminess, cohesiveness and chewiness of CSB were also evaluated. The analysis was conducted three times for each sample.

### 2.7. Microstructural Analysis

The cross-sectional structures of CSBs were observed using scanning electron microscopy (SEM) (XL-30 ESEM FEG, FEI, USA), as described previously [21]. Briefly, the CSBs were freeze dried and then fractured into pieces of 0.5 cm × 0.5 cm × 0.3 cm. The fractured CSBs were then attached to a metal disc with the natural fracture side facing upwards and then placed under vacuum conditions. The microstructures of CSBs were evaluated via SEM under an electron beam of 15 kV.

### 2.8. Analysis of Volatile Compounds

For conducting headspace solid-phase microextraction (HS-SPME), 10 g of the sample extracted from the core of the CSB was added to a 20-mL Agilent HS sample vial, and the vial was placed in a thermostat water bath at 80 °C for 40 min. The sample injector was pulled out and penetrated through the septum of the inlet rapidly and was thermally desorbed at 200 °C for 6 min in the inlet liner. Three independent replicates were performed for each sample.

The flavour components of the CSBs extracted by HS-SPME were identified using gas chromatography–mass spectrometry (GC–MS). The SPME fibre with the sample was inserted into an XL MSD spectrometer for GC–MS (Agilent Technologies, Santa Clara, CA, USA), equipped with a 123-1334 capillary column (30 m × 0.32 mm × 1.8 μm, Agilent Technologies). The GC inlet temperature was 250 °C, and the initial oven temperature was 40 °C, held for 3 min, increased to 260 °C at a rate of 10 °C/min and held for another 10 min. The carrier gas was helium at a flow rate of 1.0 mL/min. Mass spectra were recorded by electronic impact at 70 eV and an ion source temperature of 230 °C. The MS quadrupole temperature was 150 °C and the transfer line temperature was 260 °C. The mass scanning *m*/*z* was in the range of 55–500. Each *m*/*z* peak was matched with peak data in both the NIST T library (National Institute of Standards and Technology, Gaithersburg, MD, USA) (https://webbook.nist.gov (accessed on 29 November 2020)) and the Wiley Library for qualitative retrieval. The matching degree was greater than 80%. According to the peak areas of the compounds, the relative contents of the volatile components were calculated using the area normalisation method [18].

### 2.9. Sensory Quality Analysis

The sensory characteristics of CSB were evaluated referring to the method of Vicente et al. [22] with appropriate modifications. Twenty trained personnel (10 female and 10 male, aged 20 to 50) were selected to give scores on a 0 (unacceptable) to 10 (excellent) scales for appearance, flavour, taste, texture and overall acceptability of CSB samples.

### 2.10. Genome Re-Sequencing, Variant Calling and Comparative Genomic Analysis

Genomic DNA isolation and sequencing libraries were constructed and sequenced on an Illumina HiSeq 4000 instrument using 150-bp paired-end sequencing, manufactured by Novogene (Beijing, China). DNA was extracted from the *S. cerevisiae* using a commercial DNA extraction kit (Axygen, DNA gel extraction kit) following the manufacturer’s instructions. The extracted DNA was quantified using a Nanodrop spectrophotometer and its integrity was confirmed by agarose gel electrophoresis, ensuring it was suitable for sequencing. The *S. cerevisiae* S288c (sequence assembly version R64, RefSeq assembly accession: GCF 000146045.2) genome that was downloaded from RefSeq at NCBI was used as the reference genome to detect genomic variants. The Genome Analysis Toolkit (GATK v3.5) best practises pipeline was used to detect single-nucleotide polymorphisms (SNPs) and insertions/deletions (InDels). The cleaned reads were then mapped to the reference genome using BWA-mem (v0.7.13). During analysis, reads with a mapping quality ≥30 were included. Initially called SNPs were filtered with a minimum read depth of 10 and a quality score threshold of 20 [23]. Variant annotation was performed using the ANNOVAR package.

To facilitate a comparative genomic analysis, we systematically integrated phenotypic data from steamed bread fermentation experiments. Principal component analysis (PCA) was applied to reduce the dimensionality of the phenotypic data set, allowing for the selection of representative factors. These factors were then used to construct an evaluation model for scoring and ranking the fermentation performance of the yeast strains. Strains exhibiting the highest and lowest fermentation performance were identified based on their scores and subsequently subjected to comparative genomic analysis to uncover potential genetic determinants underlying their phenotypic differences [24,25]. In these two groups, if one group contained three identical mutations while the other did not contain this type of mutation, these mutations were considered different between the two groups. Gene ontology (GO) enrichment analysis was performed using the PANTHER Overrepresentation Test on the GENE ONTOLOGY RESOURCE website (http://geneontology.org/ (accessed on 20 June 2023)) [26].

### 2.11. Correlation Analysis and Establishment of a Scoring System

Correlation analysis was performed using the Performance Analytics package of the R programming language (v4.2.1, https://www.R-project.org/ (accessed on 8 July 2023)), and principal component analysis (PCA) was performed using the R packages FactoMineR and Factoextra. When establishing a scoring system, due to the different directions of hardness and other evaluation indicators, where a smaller value indicates a stronger fermentation ability of the yeast, the hardness score was multiplied by −1 to maintain consistency in data direction.

### 2.12. Statistical Analysis

All data were expressed as mean ± standard deviation from at least three independent experiments. Analysis of variance by Duncan’s test (*p* < 0.05) was conducted using SPSS v20.0 (SPSS Inc., Chicago, IL, USA). All graphs were obtained using Origin 8.5 (Origin-Lab Inc., Northampton, MA, USA).

## 3. Result and Discussion

### 3.1. Effects of Different Yeast Strains on pH, TTA and the Specific Volume of CSBs

**Figure 1:**
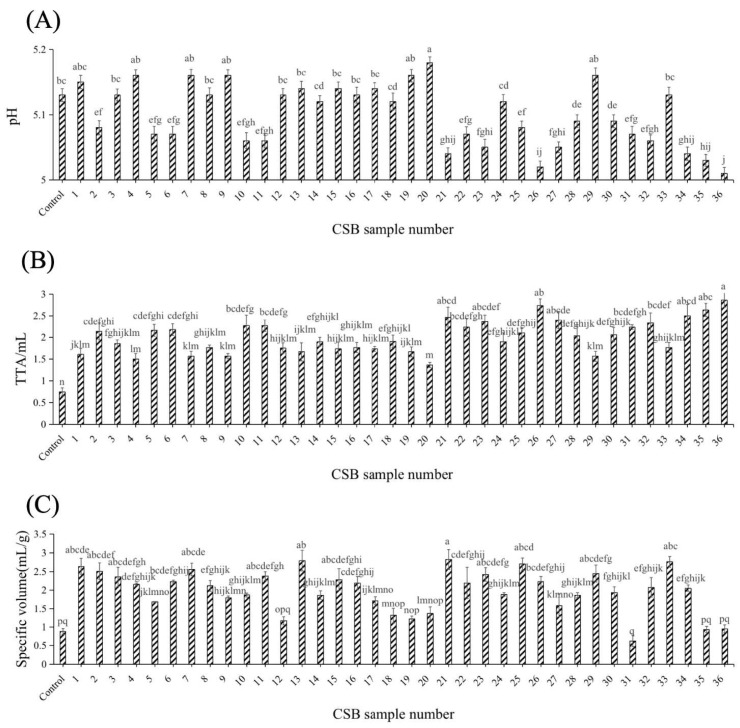
Physicochemical parameters of different CSBs. The pH (**A**), TTA (**B**), and the specific volume (**C**) of different CSBs. Error bars represent the standard deviation of at least three replicates. The column chart marked with different letters represents significant differences (*p* < 0.05).

The acidic components of CSBs are important substances that significantly impact the processing, storage, transportation and quality of CSBs [19]. Therefore, it is very necessary to measure the TTA and pH of CSBs. As shown in Figure 1A, the pH values of the 36 CSBs ranged from 5.0 to 5.2, which are acceptable. The TTA values of all of the experimental groups were significantly higher than that of the control group (Figure 1B). CSBs 26, 35 and 36 showed lower pH values and higher TTA values than the other CSBs, whereas CSBs 4, 7, 9, 20 and 29 exhibited higher pH values and lower TTA values. Fermentable sugars were utilised by yeast to produce organic acids (such as succinic acid and acetic acid) during dough fermentation, which increased the TTA value and decreased the pH value [27,28]. Li et al. [3] reported elevated succinic and acetic acid contents in fermented dough containing *S. cerevisiae* Y10 and *Torulaspora delbrueckii* Y22. In this study, CSBs 1, 2, 3, 5, 6, 8, 10–19, 21–25, 27, 28 and 30–34 exhibited moderate TTA and pH values, indicating that the corresponding *S. cerevisiae* had suitable fermentability.

Specific volume is an important index when evaluating the quality of CSB. CSBs 1, 2, 7, 13, 21, 25 and 33 exhibited significantly larger specific volumes than the control group (*p* < 0.05). Conversely, CSBs 31, 35 and 36 exhibited the smallest specific volumes (Figure 1C). A large amount of CO_2_ produced by yeast during dough fermentation acts to increase gluten expansion, improving the ability of the dough to retain air, resulting in a larger, looser and more porous structure [29]. Xu et al. [30] found that the specific volumes of *Lactobacillus sanfranciscensis* bread and *Meyerozyma guilliermondii* EH1 bread were higher than those of the other breads, indicating that the use of sourdough fermented by yeast could promote the formation of a gluten network structure and improve the fermentation capacity of dough. Our results showed that *S. cerevisiae* S1, S2, S7, S13, S21, S25 and S33 had positive effects on the network-forming properties of gluten and dough leavening ability.

### 3.2. Effects of Different Yeasts on the Textural Properties of CSBs

**Figure 2:**
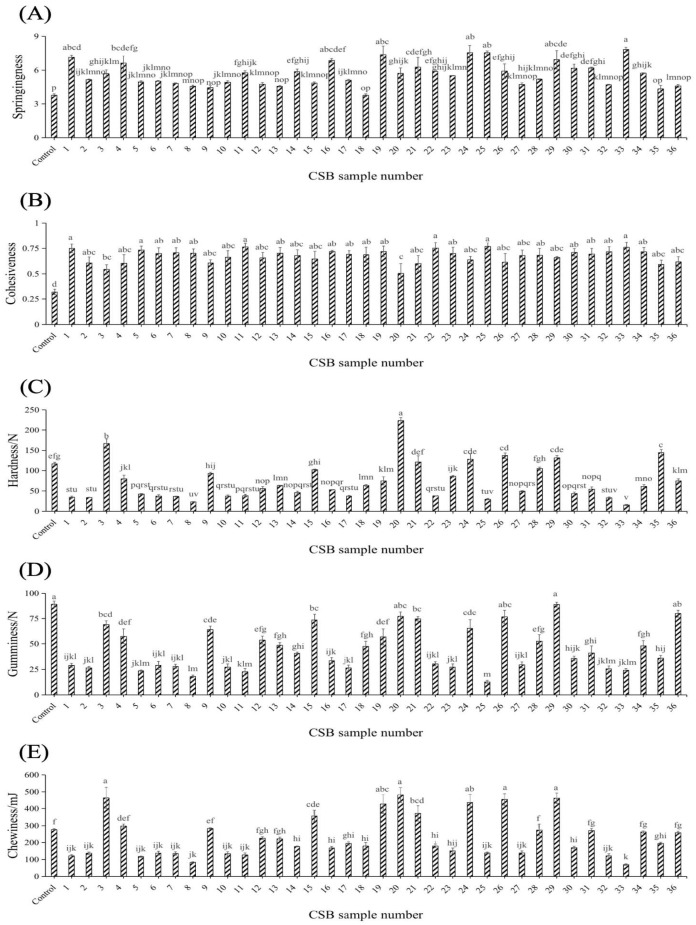
Texture analysis of different CSBs. Springiness (**A**), cohesiveness (**B**), hardness (**C**), gumminess (**D**), and chewiness (**E**) of different CSBs. Error bars represent the standard deviation of at least three replicates. The column chart marked with different letters represents significant differences (*p* < 0.05).

Good quality CSB exhibits moderate textural properties. Springiness and cohesiveness were positively correlated with CSB quality, while hardness, gumminess and chewiness were negatively correlated with CSB quality [20]. In addition, this study showed that the springiness and cohesiveness values of all of the experimental groups were significantly higher than those of the control group; CSBs 1, 19, 24, 25 and 33 showed higher springiness values (7.13 ± 0.21, 7.37 ± 0.78, 7.55 ± 0.64, 7.55 ± 0.16 and 7.83 ± 0.22, respectively) than the other CSBs (Figure 2A). CSBs 1, 11, 22, 25 and 33 showed higher cohesiveness values (>0.75) than the other CSBs (Figure 2B). Based on the comprehensive consideration of springiness and cohesiveness, *S. cerevisiae* S1, S25 and S33 might exhibit superior fermentative activity, allowing them to produce more CO_2_, which increases the pore size in CSBs. This promotes the gas-holding ability of CSB gluten and maintains the intermolecular attraction between the CSB ingredients, thereby reducing water loss and making the CSB soft and elastic and thus appealing to consumers [31,32]. As shown in Figure 2C–E, CSBs 8, 25 and 33 exhibited lower hardness (23.37 ± 0.85 N, 29.90 ± 1.84 N and 15.73 ± 1.34 N, respectively) and gumminess values (18.00 ± 1.22, 12.70 ± 1.54 and 24.20 ± 1.55, respectively), whereas CSBs 8 and 33 had lower chewiness values (82.34 ± 4.14 and 69.92 ± 4.64, respectively). Yeast with strong fermentation power can convert starch into low molecular weight saccharides, reducing the amount of available starch for retrogradation, which could improve the softness and reduce the hardness of CSB [33]. Gumminess indicates cohesion or stickiness and is correlated to hardness. Chewiness has a certain correlation with hardness and springiness. Li et al. [34] found that the weakening of the starch–protein network and the enhancement of the gas-holding capacity during yeast fermentation decrease the hardness, gumminess and chewiness of CSB. *S. cerevisiae* S1, S8, S25 and S33 are the best yeasts for preparing CSB.

### 3.3. Effects of Different Yeast Strains on CSB Microstructure

Yeasts with a strong fermentation capacity will produce more CO_2_ during the fermentation process, thus improving the specific volume of the CSB [28]. As depicted in Figure 1C, CSBs 1, 25 and 33 exhibit high specific volume, while CSBs 12, 35 and 36 display low specific volume. Additionally, CSBs 14, 16, 17 and 30 possess medium specific volume. Based on previous experiments, 10 *S. cerevisiae* (S1, S12, S14, S16, S17, S25, S30, S33, S35 and S36) with strong, medium and weak fermentation abilities were selected for further evaluation.

**Figure 3:**
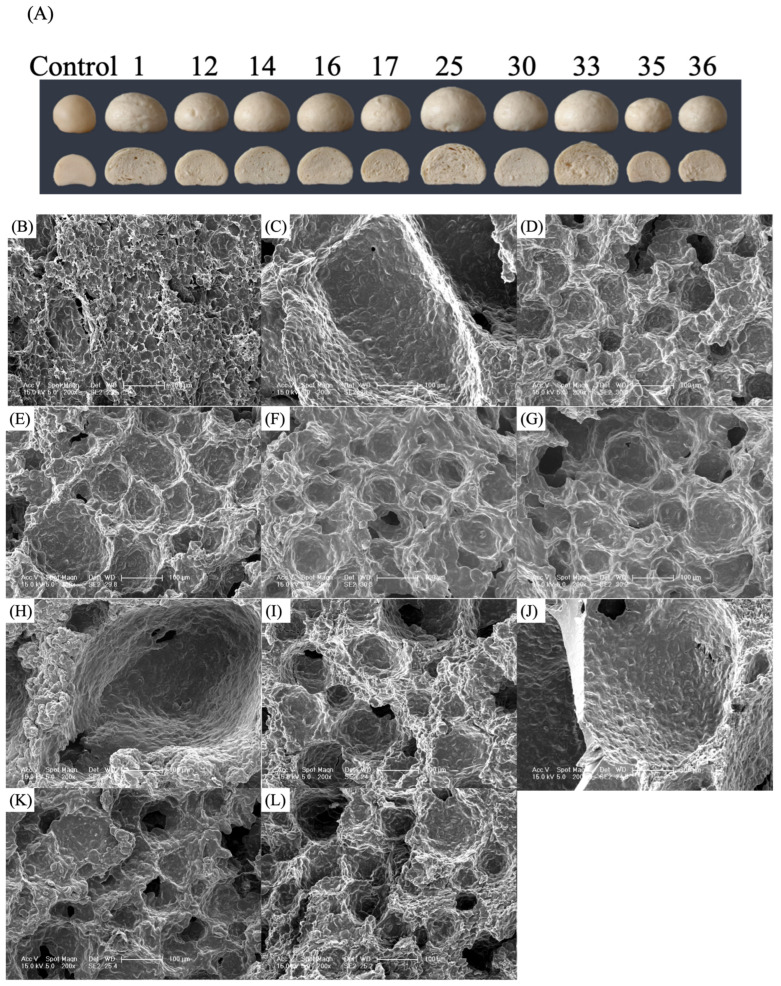
Appearance and microstructures of different CSBs. Appearance of different CSBs (**A**). The microstructure of CSB prepared without yeast (**B**), and microstructures of CSBs prepared with *S. cerevisiae* S1 (**C**), S 12 (**D**), S 14 (**E**), S 16 (**F**), S 17 (**G**), S 25 (**H**), S 30 (**I**), S 33 (**J**), S 35 (**K**), and S 36 (**L**), respectively.

The microstructure of CSB affects its macroscopic characteristics (specific volume) and sensory qualities (such as elasticity, viscosity and taste). The microstructures of CSBs were analysed via SEM (Figure 3). No continuous and smooth gluten network structure was formed in the CSB prepared without yeast, and starch particles were exposed, contributing to the tight and hard structure of the CSB (Figure 3A). A loose and porous structure with large pores was observed in CSBs 1, 25 and 33, and starch particles were wrapped in the network structure of gluten proteins (Figure 3B,G,I). CSBs 12 and 30 (Figure 3C,H) had abundant smaller cavities with rough morphology, indicating that some starch particles were not well wrapped by the gluten network. CSBs 14, 16 and 17 (Figure 3D–F) showed smaller voids with smooth morphology and fewer breaks of the gluten structure. CSBs 35 and 36 showed a tight gluten network structure with small holes, and some starch granules were exposed on the gluten protein surface (Figure 3J,K), Yeast with good fermentation capacity might produce more CO_2_, creating a conducive environment for starch hydration, thus promoting gluten proteins to wrap starch particles more tightly and ensuring that the gluten network structure with larger pores and higher stability [21]. Therefore, *S. cerevisiae* S1, S25 and S33 had better fermentation capacity than other *S. cerevisiae*, which was consistent with the results of the previous analysis of specific volume and texture.

### 3.4. Identification of Volatile Compounds in CSBs

**Figure 4:**
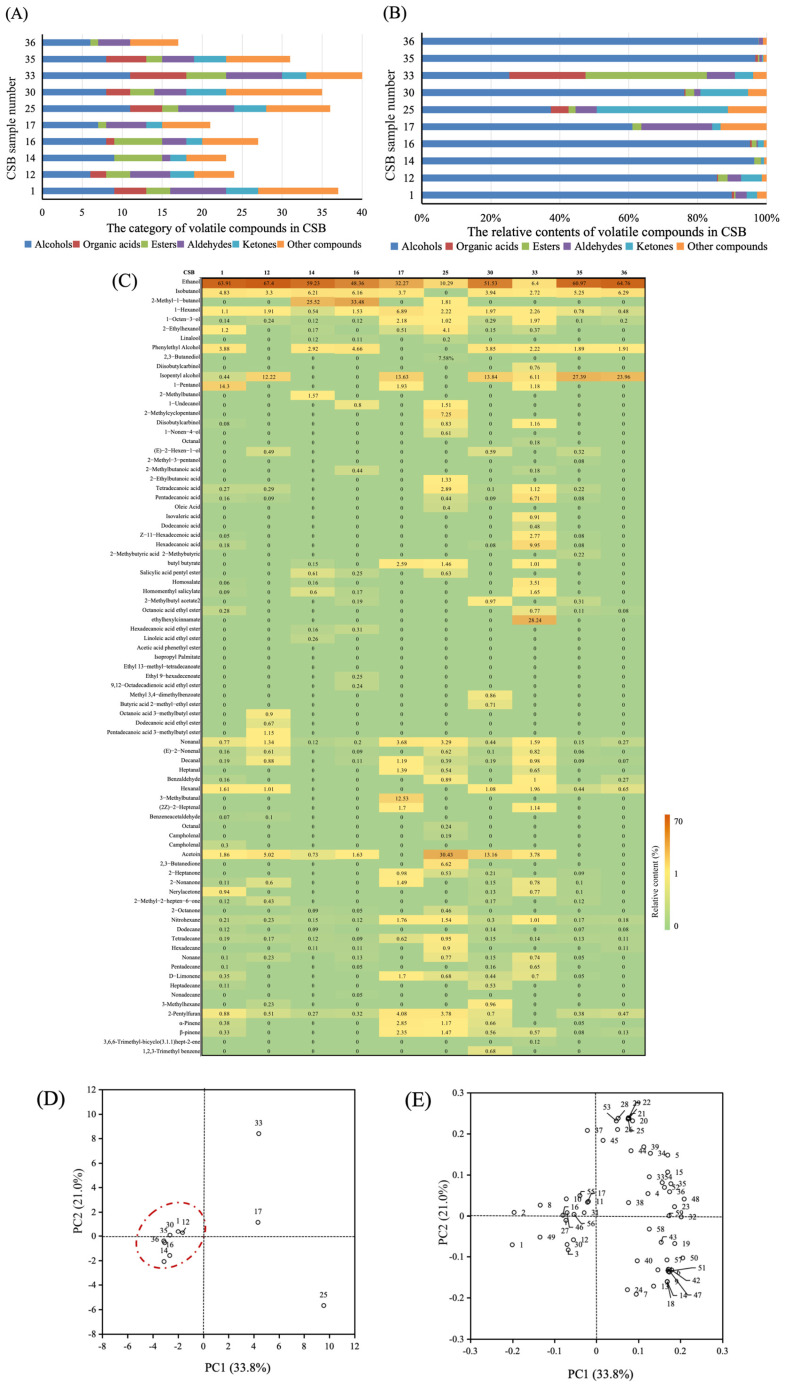
Volatile compound analysis of CSBs fermented by 10 different *S. cerevisiae*. The category (**A**) and relative contents (**B**) of volatile compounds in CSBs, the volatile compounds identified in different CSBs (**C**), the score plot of CSB samples (**D**), and the loading chart of volatile compounds in CSBs (**E**). (1–59 corresponds to the volatile compounds in the top to bottom order shown in (**C**).

Volatile flavour substances are a crucial factor affecting consumers’ acceptance and the overall quality of CSBs [18]. In this study, 59 volatile compounds, comprising 16 alcohols, six acids, nine esters, nine aldehydes, seven ketones and 12 other compounds (Figure 4A,C), belonging to six categories were identified and quantified in 10 CSBs. Among them, alcohols had the highest average proportion, accounting for 76.12%. Among alcohols, ethanol was the most important, accounting for an average of 46.51%. This result was consistent with Pico’s report that the most abundant compound in yeast-fermented bread was alcohol [35]. The contents of other volatile compounds were similar, among which the acid content was the lowest, accounting for 2.84% (Figure 4B,C). Liu et al. [36] identified 41 compounds, comprising 11 alcohols, 11 aldehydes, five ketones, nine acids, three esters and two other compounds, in the CSBs fermented by five yeast species. Although the categories of volatile compounds are the same, the total number of compounds are less than those observed in this study. The CSBs’ flavour was different due to the different threshold values and relative contents of some volatile substances [37]. In this study, 34, 21, 22, 24, 21, 33, 32, 39, 29 and 17 volatile compounds were found in CSBs 1, 12, 14, 16, 17, 25, 30, 33, 35 and 36, respectively (Figure 4A). Moreover, CSBs 25 and 33 contained a wide variety and a more balanced content of volatile compounds, indicating that *S. cerevisiae* S25 and S33 could contribute to the abundant and harmonious flavour of CSBs. The aroma threshold value of alcohols is high, but the alcohol content in CSB is also high; therefore, it greatly contributes to the aroma of CSBs. Notably, 2-methyl-1-butanol, which has a balsamic, alcoholic and malty aroma, was dominant and positively correlated with the aroma of CSBs [38]. In addition, 1-heptanol has a fruity and flowery aroma, phenylethyl alcohol has a rose-honey-like aroma and 1-octene-3-ol has mushroom and liquorice aromas [39]. The production of some carboxylic acids may be related to the fatty and amino acid biosynthetic pathways in yeast. Tetradecanoic, pentadecanoic and hexadecanoic acids were the main components, and they had special fragrances that played a positive role in improving CSB flavour [39]. Esters are an important aromatic component in CSBs due to their low aroma threshold, and they have various pleasant and fruity fragrances [40]. Strain-specific esters formed by yeast do not directly improve the quality of bread but contribute to the unique flavour of individual products, ultimately affecting their final quality [41]. Octanoic acid ethyl ester has a brandy, fruity, rose, orange and cool fragrance at low concentrations [18]. In the CSB 33, ethylhexylcinnamate constituted the highest proportion (28.24%), a finding that diverged from earlier reports indicating that hexyl acetate and phenethyl acetate would be produced in greater quantities when yeast metabolism was more active [37,40]. This outcome implies that *S. cerevisiae* S33 can be utilized to create fermented foods with novel and innovative taste profiles. Aldehydes can significantly increase the flavour of CSB because of their lower threshold values. Hexanal and heptanal are produced by the oxidation of unsaturated lipids during CSB fermentation, and hexanal, which can be used as a food spice, has a strong, sweet and fruity taste [42]. Nonanal has a rose and citrus aroma [43]. Ketones are formed by the catalytic oxidation of lipids by lipoxygenase [44]. Acetoin was the dominant ketone in all of the CSBs, and its proportion was the highest in CSB 25 (30.43%), followed by those in CSBs 30 (13.66%), 12 (5.17%) and 33 (3.78%). Birch et al. [45] reported that acetoin and 2, 3-butanedione, which are the important aroma compounds in bread crumb, were synthesised from α-acetolactate secreted by yeast cells in a non-enzymatic reaction. The aroma activity of acetoin was not obvious because of its high odour threshold (800 μg/kg).

To further identify the difference in the flavour characteristics of each sample, the flavour profiles were analysed via PCA (Figure 4D,E). Figure 4D shows the score analysis of CSBs, which is a tool for visualising the sample properties and variable relationships among samples [46]. The total variance contribution rates of PC1 (33.8%) and PC2 (21.0%) were 54.8%, suggesting that the two principal components could reveal the difference in the flavour profiles of CSBs. CSBs 17, 25 and 33 were distributed in distinctly separate regions, whereas CSBs 1, 12, 14, 16, 30, 35 and 36 were distributed in similar regions, indicating that CSBs 17, 25 and 33 could be distinguished from other CSBs by PCA. Figure 4E shows the loading analysis of CSBs, which could explain the influence of different yeasts on the volatile compounds of CSBs. 1-Hexanol and 3-methylbutanal significantly contributed to the flavour of CSB 17. The flavour compounds of CSB 25 mainly included 2-ethylhexanol, 2,3-butanediol, 2,3-butanedione, 2-octanone and hexadecane. The flavour substances that had a high correlation with CSB 33 were pentadecanoic acid, hexadecanoic acid, Z-11-hexadecenoic acid, homosalate, homomenthyl salicylate, octanoic acid ethyl ester, ethylhexyl cinnamate and pentadecane. It can be seen that CSB 33 contains more characteristic flavour substances, which is consistent with the results shown in Figure 4C. Thus, in terms of flavour components, *S. cerevisiae* S25 and S33 offer greater advantages in terms of improving the quality of CSB.

### 3.5. Sensory Analysis

**Figure 5:**
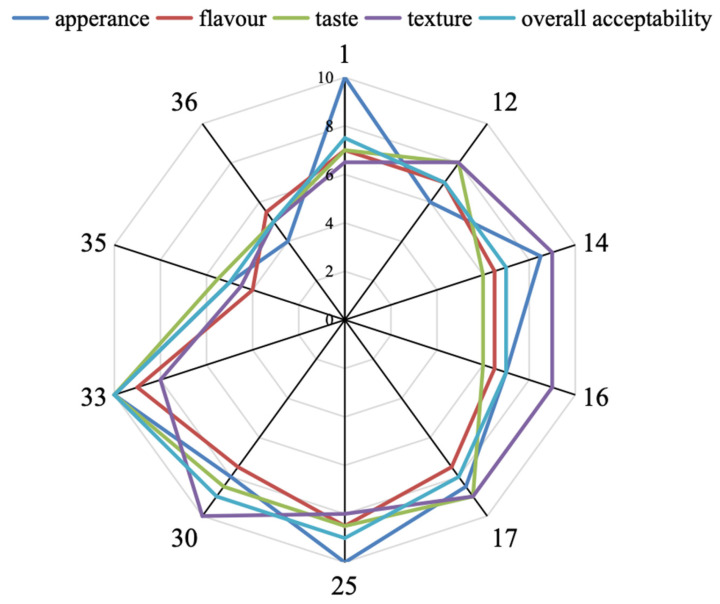
The sensory evaluation of CSBs fermented by 10 different *S. cerevisiae*.

Sensory analysis of CSB was conducted to more accurately assess the product’s acceptability among consumers. Producing high-quality CSB requires all of the ingredients to work synergistically to achieve an overall balance of flavour and mouthfeel, and the sensory differences of the CSBs were mainly reflected in five sensory indicators such as appearance, flavour, taste, texture and overall acceptability. Based on the physicochemical indexes and flavour characteristics of the CSBs, a multi-dimensional analysis and statistical evaluation of the CSBs were conducted. As shown in Figure 5, CSB33 had the highest evaluation scores in appearance, flavour, taste, texture and acceptability, followed by CSB25 and 1. Meanwhile, CSB35 and 36 had the lowest scores. The results were consistent with those previously stated.

### 3.6. Comparative Genomic Analysis

**Figure 6:**
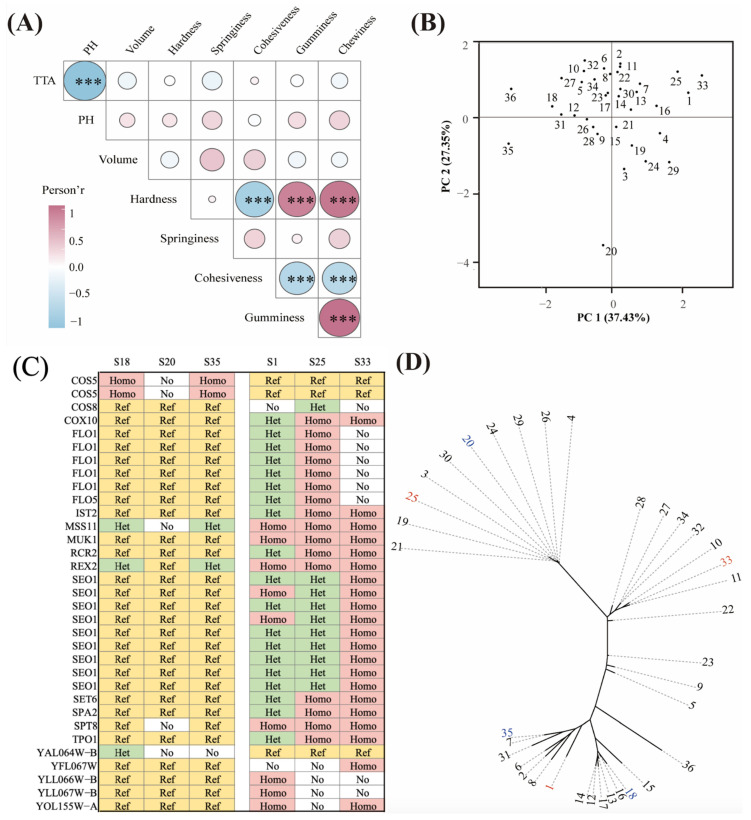
The comparative genomic analysis of 36 *S. cerevisiae*. The correlation map of physical parameters of CSB produced (**A**). In the figure, red indicates a positive correlation, while blue indicates a negative correlation. The size of the circles represents the absolute value of the Pearson correlation coefficient. Significant correlation test results (*** *p* < 0.05) are annotated within the circles. The figure was generated using the R package LinkET. PCA of 36 strains based on physical parameters of CSB produced (**B**). The figure is generated using the R package FactoMineR. The figure displays information on non-synonymous mutation sites in genes that show significant differences among the top and bottom three strains (**C**). The colours represent the comparison results between the analysed strains and the reference genome R64. Yellow indicates that the specific site is consistent with the reference genome, red indicates that both alleles at the specific site are inconsistent with the reference genome, green indicates a heterozygous state where one allele is consistent and the other is inconsistent with the reference genome, and “No” indicates that the sequencing data quality at the specific site is poor. The phylogenetic tree of 36 strains (**D**). In the tree, strains marked in blue are the selected strains with strong fermentation capabilities, while those marked in red are strains with weaker fermentation capabilities. The phylogenetic tree is based on whole-genome SNPs and was inferred using IQtree, with visualization performed using iTOL.

As shown in Figure 6A and Figure S1, pH was significantly correlated with TTA, with a correlation coefficient of 0.99, and any two of the four indicators (hardness, cohesiveness, gumminess and chewiness) showed a good positive correlation, with a correlation coefficient of 0.6–1.0, indicating that these indicators could be used interchangeably. Finally, the TTA, volume, hardness and springiness were retained as four evaluation indicators to perform a PCA analysis.

To establish a scientific evaluation system for CSBs and comprehensively evaluate the indicators, PCA was used to analyse and reduce the four existing evaluation indicators. Based on the clustering results, two principal components (1.45 and 1.06) with eigenvalues greater than 1 were selected, which could explain 64.78% of the evaluation indicators. The strains distributed in the first and fourth quadrants represent strains with strong and weak fermentation abilities, respectively (Figure 6B). Through the study of Cos2 (Cos2 for the variables in PCA analysis), a linear formula was constructed using different principal components to represent the variables:

y = 1.45 (0.35TTA + 0.57Volume + 0.02Hardness + 0.55Springiness + 1.06 (0.17TTA + 0.09Volume + 0.81Hardness + 0.03Springiness)

y = 0.68TTA + 0.93Volume + 0.89Hardness + 0.84Springiness

where y represents different fermentation capacity scores.

According to the formula, fermentation ability scores are presented in Table 1. *S. cerevisiae* S1, S25 and S33 exhibited strong fermentation ability, which was consistent with the results of the above experiments.

Through comparative genomic analysis, we explored the genes related to the fermentation ability of CSBs. According to the experimental data, *S. cerevisiae* S1, S25 and S33 were three strains with stronger fermentation abilities, whereas *S. cerevisiae* S18, S20 and S35 were three strains with weaker fermentation abilities, consistent with the scores presented in Table 1. In the comparison of the genomes of these two groups with large phenotypic differences, some loci differed, involving 169 genes and 366 variations.

GO enrichment analysis was conducted on the 169 genes, and the results showed that the genes with different variations in the two groups of strains with strong and weak fermentation abilities were roughly enriched by oxidative phosphorylation, electron transfer and cell respiration. During CSB fermentation, yeast cells use sugars, amino acids, proteins and other substances as substrates, producing alcohols, acids, esters, aldehydes, ketones and some flavour substances. CO_2_ is also released during the fermentation process, affecting the volume of the CSB (Table 2).

The heatmap showed 33 differences in non-synonymous mutations between the three strains with strong fermentation ability and the three strains with weak fermentation ability, involving 19 genes (Figure 6C). These mutations exhibited completely different variation states in the two groups of yeast. *COS5*, *COS8* and *COX10* are mitochondrial genes. As Marr et al. [47] described, *COS5* has an interaction with the Hxt9 hexose transporter, which may impact Hxt9 turnover, and *COS8* relates to NH^4+^ transport. These two genes are highly correlated with the assimilation of substrates and the metabolism of flavor-related compounds, not only in the wine mentioned in the article but also in CSB fermentation. *COX10* may help control hemeosynthesis in *S. cerevisiae* [48]. *FLO1* and *FLO9* are involved in cell aggregation, and mutations may change the oxygen transport in cells. These three genes are involved in cell respiration. Cellular respiration is the foundation of dough fermentation, as described in He et al. [49]. *MSS11* is a transcription factor involved in starch metabolism, which regulates the use of starch during fermentation [50]. SPT8 is a general transcription regulatory factor required for all regulated transcription [51]. The heatmap also showed that many variations involved *SEO1*, a transport protein of sulphur-containing compounds. The relationship between *SEO1* and the fermentation of brewing yeast is currently unclear, but a comparative genomic analysis of multiple groups related to alcohol fermentation showed that *SEO1* has different genotypes in different strains [52,53]. Therefore, *SEO1* may be involved in the production of flavour compounds during the fermentation process. Most of the genes mentioned above have been studied in other fermentation research, such as work on wine fermentation. This likely involves the fundamental aspects of fermentation—substrate metabolism and cellular respiration. Similar studies are also applicable to other fermented products, such as beer, bread and yogurt. In these products, the optimization of substrate metabolism and cellular respiration is equally important, as they directly affect the flavour, texture and shelf life of the products. By comparing the gene expression patterns in different fermented products, commonalities and specificities can be identified, providing references for cross-domain fermentation research.

The phylogenetic tree illustrated that the two groups of strains with different performances were distributed in different clades of the unrooted tree and did not cluster together (Figure 6D). This indicates that these variations occurred during different evolutionary events and did not originate from the same evolutionary ancestor.

## 4. Conclusions

In this study, the relationship between the gene composition and various phenotypes of *S. cerevisiae* and the quality of CSB during fermentation was investigated. CSBs 1, 25 and 33 exhibited modest pH and TTA values, relatively high springiness and cohesiveness values and relatively low hardness, gumminess and chewiness values. The micromorphology analysis showed that *S. cerevisiae* S1, S25 and S33 contributed to the formation of a loose and porous gluten network structure with large pores in CSBs. The results of volatile component analysis showed that *S. cerevisiae* S1, S25 and S33 gave CSBs a distinctive and harmonious flavour through the coordination of species, relative substance content and odour threshold of volatiles. Comparative genomic analysis showed that the strains with strong (S1, S25 and S33) and weak fermentation abilities (S18, S20 and S35) had 169 genes and 366 variations. These data provide a preliminary experimental basis for further research and applications of *S. cerevisiae* S1, S25 and S33 in grain-based fermented foods.

## Figures and Tables

**Table 1:** The score of different strains in CSB fermentation.

Strain ID (S)	TTA	Volume	Hardness	Springiness	Score
20	−1.7331	−1.1101	−3.2478	0.0645	−130.0260
35	1.6519	−1.8862	−1.5551	−1.2485	−78.8627
18	−0.2989	−1.2108	0.1840	−1.8004	−68.8262
9	−1.1986	−0.3400	−0.4485	−1.1439	−64.0432
12	−0.7086	−1.4656	0.3489	−0.8521	−57.9189
3	−0.4236	0.6494	−2.0242	0.0391	−37.3833
15	−0.7532	0.5190	−0.6427	−0.7443	−31.4819
28	0.0481	−0.2215	−0.7005	−0.4271	−29.7159
31	0.5830	−2.4312	0.3796	0.5244	−28.1468
36	2.2666	−1.8506	−0.0587	−0.9916	−27.4244
17	−0.7532	−0.4822	0.7423	−0.5064	−18.6429
19	−0.9314	−1.3767	−0.0523	1.6503	−15.0142
27	1.0284	−0.7251	0.4910	−0.8711	−6.8956
5	0.3870	−0.5414	0.6374	−0.6491	−5.5825
29	−1.1986	0.8093	−1.2760	1.2221	−4.4979
8	−0.6641	0.2228	1.0457	−1.0138	−4.1362
4	−1.3768	0.2939	−0.1594	0.9335	−0.6060
13	−0.9314	1.4195	0.1940	−1.0234	0.1765
24	−0.3078	−0.1860	−1.2074	1.8215	1.6870
7	−1.1986	0.9989	0.7666	−0.7696	3.9897
14	−0.3078	−0.2215	0.5645	0.2231	7.0370
10	0.6721	−0.2156	0.7452	−0.6777	9.0823
26	1.9192	0.4183	−1.39233	0.248439	17.06984
32	0.8502	0.1517	0.832254	−0.89015	18.43684
6	0.4315	0.4242	0.740158	−0.573	22.36366
30	0.1198	−0.0971	0.610938	0.502165	24.57858
23	0.9393	0.7619	−0.29574	−0.12263	25.30741
16	−0.6641	0.3531	0.408185	1.165022	31.2823
34	1.2956	0.1162	0.228276	0.080346	32.42902
2	0.3157	0.9219	0.817261	−0.45248	36.63929
22	0.5830	0.3591	0.735874	0.242096	40.89846
21	1.2065	1.4787	−1.05178	0.597312	45.35632
11	0.6810	0.6849	0.724452	0.112062	47.37149
1	−1.0917	1.1470	0.800841	1.421919	57.37611
33	−0.6641	1.3662	1.209204	2.087948	93.81723
25	0.2267	1.2655	0.905788	1.821536	94.30497

**Table 2:** The GO enrichment of genes detected in comparative genomics.

GO Biological Process Complete	Fold Enrichment	*p*-Value	FDR
Electron transport coupled proton transport (GO:0015990)	53.07	7.34 × 10^−6^	2.32 × 10^−3^
Mitochondrial mRNA processing (GO:0090615)	23.59	6.98 × 10^−5^	1.68 × 10^−2^
Mitochondrial electron transport, cytochrome c to oxygen (GO:0006123)	15.61	4.06 × 10^−5^	1.03 × 10^−2^
Mitochondrial ATP synthesis coupled electron transport (GO:0042775)	12.25	1.69 × 10^−7^	2.13 × 10^−4^
ATP synthesis coupled electron transport (GO:0042773)	12.25	1.69 × 10^−7^	1.71 × 10^−4^
Aerobic electron transport chain (GO:0019646)	12.25	1.69 × 10^−7^	1.42 × 10^−4^
Respiratory electron transport chain (GO:0022904)	11.11	3.48 × 10^−7^	2.20 × 10^−4^
Oxidative phosphorylation (GO:0006119)	10.61	4.88 × 10^−7^	2.74 × 10^−4^
Electron transport chain (GO:0022900)	10.41	1.29 × 10^−7^	2.17 × 10^−4^
Proton transmembrane transport (GO:1902600)	7.24	2.40 × 10^−7^	1.73 × 10^−4^
Aerobic respiration (GO:0009060)	5.9	4.89 × 10^−6^	1.65 × 10^−3^
Cellular respiration (GO:0045333)	5.56	8.21 × 10^−6^	2.30 × 10^−3^
Inorganic ion transmembrane transport (GO:0098660)	5.21	5.41 × 10^−8^	2.73 × 10^−4^
Inorganic cation transmembrane transport (GO:0098662)	4.82	2.12 × 10^−6^	9.75 × 10^−4^
Cation transmembrane transport (GO:0098655)	4.31	1.51 × 10^−6^	7.63 × 10^−4^
Ion transmembrane transport (GO:0034220)	4.25	7.86 × 10^−8^	1.99 × 10^−4^
Energy derivation by oxidation of organic compounds (GO:0015980)	3.97	1.46 × 10^−4^	3.08 × 10^−2^

## Data Availability

Data will be made available on request.

## Supplementary Materials

The following supporting information can be downloaded at: https://www.mdpi.com/article/10.3390/jof11020114/s1, Figure S1: The comparative genomic analysis of 36 *S. cerevisiae*. The correlation map of physical parameters of CSB produced (*** indicates significantly different *p* < 0.0005).
