# Supplementary material for: Comparative Genomics and Characterisation of the Role of Saccharomyces cerevisiae Respiration in the Fermentation of Chinese Steamed Bread
Source: J Fungi (Basel). 2025 Feb 3;11(2):114. doi: 10.3390/jof11020114 (PMC11856702; doi:10.3390/jof11020114)
Supplement: Supplementary file 1 [file jof-11-00114-s001.zip › jof-3401684-supplementary.pdf]

Supporting Information

Comparative Genomics and Characterisation of the Role of *Saccharomyces cerevisiae* Respiration in the Fermentation of Chinese Steamed Bread

Yawen Gao [1], Yufeng Guo [2, 3], Jianing Pang [1], Mingkai Liu [4], Tengdan Yuan [1], Qinhong Wang [2,3,*] and Jingsheng Liu [1*]

[1] College of Food Science and Engineering, Jilin Agricultural University, Changchun 130118, P.R. China.

[2] Key Laboratory of Engineering Biology for Low-carbon Biosynthesis, Tianjin Institutes of Industrial Biotechnology, Chinese Academy of Science, Tianjin 300308, P.R. China.

[3] National Center of Technology Innovation for Synthetic Biology, Tianjin 300308, P.R. China.

[4] College of Food Science and Engineering, Qilu University of Technology (Shandong Academy of Science), Jinan 250353, P.R. China.

***** Correspondence: Qinhong Wang, 32 West 7th Avenue, Tianjin Airport Economic Area, Tianjin, 300308, P. R. China. Tel: +86-22-84861950; Fax: +86-22-84861996; E-mail: wang_qh@tib.cas.cn; Jingsheng Liu, No. 2888 Xincheng Street, Jingyue District, Changchun,130118, P. R. China. Tel: +86-13504705959; E-mail: liujingsheng@jlau.edu.cn

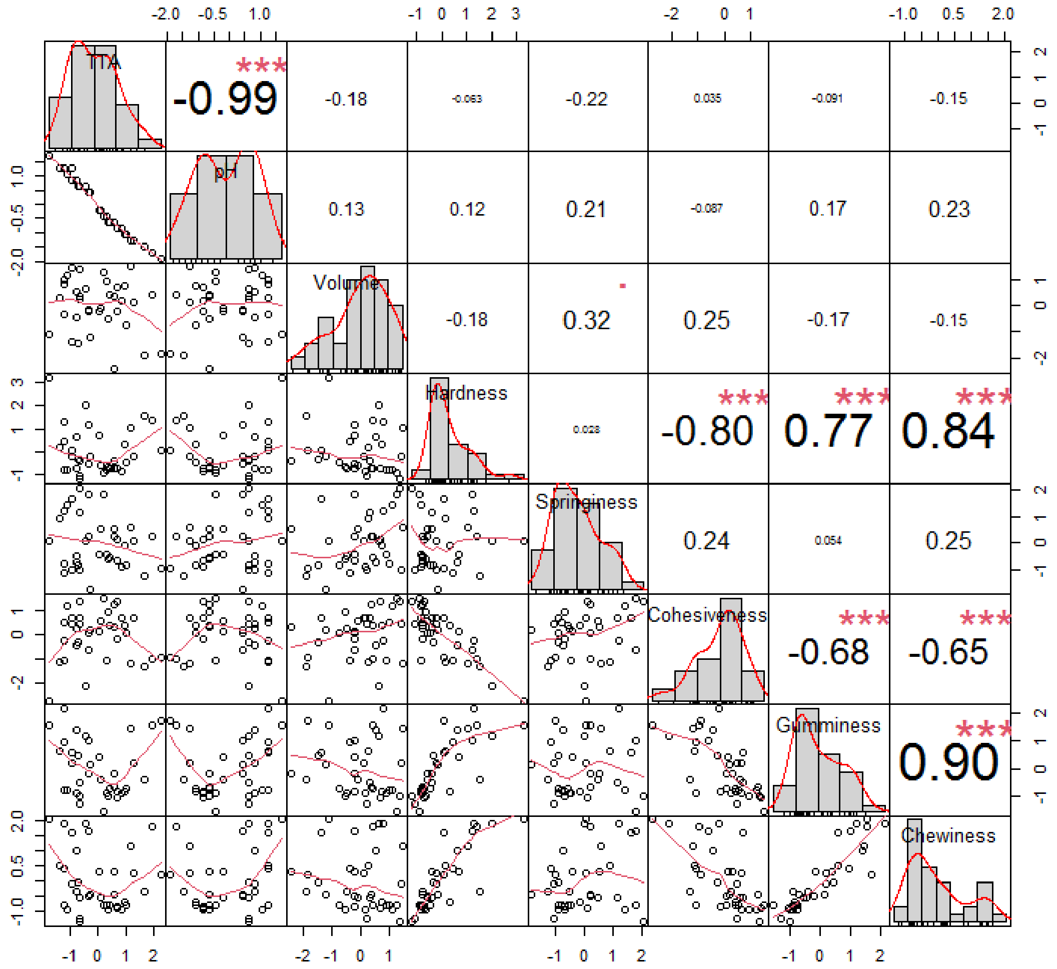

Figure S1. The comparative genomic analysis of 36 *S. cerevisiae*. The correlation map of physical parameters of CSB produced (*** indicates significantly different P<0.0005).
